# Pre-RC Protein MCM7 depletion promotes mitotic exit by Inhibiting CDK1 activity

**DOI:** 10.1038/s41598-017-03148-3

**Published:** 2017-06-06

**Authors:** Dianpeng Zheng, Sichao Ye, Xiuyun Wang, Yongjun Zhang, Daoyu Yan, Xiangsheng Cai, Weihong Gao, Hongbo Shan, Yang Gao, Juanjuan Chen, Zhiming Hu, Hongwei Li, Jinlong Li

**Affiliations:** 1 Institute of Biotherapy, School of Laboratory Medicine and Biotechnology, Southern Medical University, Guangzhou, Guangdong China; 2 Department of Endoscopy, Sun Yat-sen University Cancer Center, Guangzhou, China; 3 State Key Laboratory of Oncology in South China and Collaborative Innovation Center for Cancer Medicine, Sun Yat-sen University Cancer Center, Guangzhou, China

## Abstract

MCM7, a subunit of mini-chromosome maintenance proteins (MCM) complex, plays an important role in initiating DNA replication during the G1 phase and extending DNA strands during the S phase. Here, we demonstrated that MCM7 is not only sustained but maintains association with chromatin during M phase. Remarkably, MCM7 siRNA can accelerate mitotic exit. MCM7 depletion leads to CDK1 inactivation and promotes subsequent cohesin/RAD21 cleavage, which eventually leads to sister chromatin segregation. Moreover, MCM7 is co-localized with tubulin in the mitotic cells and MCM7 depletion results in aberrant mitosis. Our results indicate that MCM7 may exert certain functions on spindle formation to prevent cytokinesis during early mitosis by regulating CDK1 activity.

## Introduction

Eukaryotic DNA replication is a methodical and strictly controlled process evolved to ensure accurate replication. Many mistakes that occur during DNA replication can cause genomic instability. The pre-replicative complex (Pre-RC), which plays an important role in initiating DNA replication, consists of the origin recognition complex (ORC), CDC6, CDT1 and mini-chromosome maintenance proteins (MCMs). MCM2-7 is a heterohexamer complex which contains six MCM homologues. MCMs are determined to be DNA helicases involved in the initiation of DNA replication. MCM subunits are evolutionarily conserved in organisms ranging from yeast to human including features such as the ATPase motifs^1, 2^. Recent studies have determined that there are more MCMs bound onto chromatin than required by replication origins, and the phenomenon is coined as the “MCM paradox”. The mechanism of the paradox is still unknown. Recent cancer studies have also suggested that MCM2-7 complex overexpression is relative to proliferation and malignancy of various cancers^3–12^.

MCM7, a key component of the MCM complex, forms a double trimeric complex with MCM4 and MCM6. The double trimeric complex initiate DNA replication through unwinding DNA double strand. During S phase, the complex participates in elongating DNA stands. However, a growing body of evidence has demonstrated that the function of MCM7 is not limited to starting DNA replication. Recent studies suggest that MCM7 interact with the polo-box domain (PBD) of endogenous PLK1, a kinase expressed during S, G2 and M phases of the cell cycle and plays an important role in dissociating the cohesion from chromosome^13^. MCM7 also interact with ATR-interacting protein (ATRIP)-ATR, which is activated in the presence DNA replication damage^14^. Furthermore, the MCM7 gene codes for an important microRNA cluster miR-106b-25, and contribute to the induction of megakaryocytes polyploidy^15^. *In vitro*, MCM7 is highly expressed in multiple malignancies. Immunohistochemical studies in a variety of tissues show increased expression of MCM7 in several solid tumors, including melanoma^16^, endometrial carcinoma^17^, esophageal adenocarcinoma^18^, oral squamous cell carcinoma^19^, colorectal adenocarcinoma^20^, glioblastoma^21^ and thyroid cancer^22^.

In this study, mRNA copy number and expression of MCMs were examined during cell cycle progression. We have also explored the correlation between MCM7 depletion and the M phase process. From this we have discovered that MCM7 depletion accelerates the M phase by promoting CDK1 phosphorylation and cleaving Cohesin/RAD21, allowing sister chromatids to segregate.

## Results

### MCMs’ mRNA express in a cell cycle

**Figure 1:**
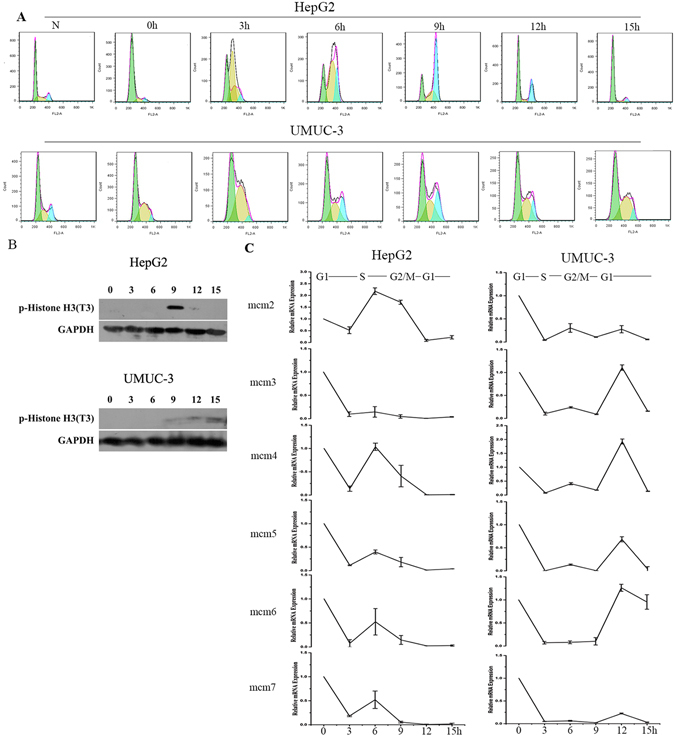
MCMs’ mRNA express in HepG2 and UMUC-3 Cells. (**A**) Cells were synchronized with double thymine and incubated in fresh medium for 0 h, 3 h, 6 h, 9 h, 12 h and 15 h. Cell cycle was analyzed by FACS. Data are shown as mean ± SD of three independent experiments. N: asynchronized cells. (**B**) P-Histone H3 was detected by western-blot. (**C**) MCMs’ mRNA levels were examined by real-time RCR. Data are normalized to the beta-actin level in each sample. These experiments were performed in triplicate, and the values are expressed as mean ± SD.

To identify the mRNA expression levels of MCMs in a single cell cycle, HepG2 cells and UMUC-3 cells were synchronized at G1 phase by double thymidine block. Cells were then incubated in fresh medium and collected at 0 h, 3 h, 6 h, 9 h, 12 h, and 15 h. HepG2 cells were successfully synchronized in G1 phase (G1: 86.34% ± 0.62% at 0 h) and slowly entered the S phase at 6 h. After incubation in fresh medium at 9 h, most cells progressed into the G2/M phase (G2/M: 60.30% ± 2.93%) and entered the next cell cycle by the 15 h timepoint. Compared to HepG2, UMUC-3 demonstrated quicker cell cycle progression. UMUC-3 were arrested in G1 (G1: 63.11% ± 2.13%) at 0 h. After incubation in fresh medium, at about 6 h, most cells had entered the G2/M phase (G2/M: 40.61% ± 3.87%) and entered the next cell cycle after 9 h (Fig. 1A).

To confirm the cell cycle progression of the above cells, the mitotic marker p-Histone (Thr3) was examined by Western Blotting. In HepG2 cells, p-histone H3 was detected after releasing 9 h and deceased after releasing 12 h, which was consistent with FCM result. Similar results were observed in UMUC-3 cells (Fig. 1B).

To determine MCMs’ mRNA expression in one cell cycle, MCMs’ mRNA isolated in these time points were detected by QPCR (Fig. 1C). We found that all the six MCMs’ mRNA gradually decreased during G2/M phase (HepG2: 9 h and UMUC-3: 6 h–9 h). Interestingly, there was slight increase of MCMs’ mRNA during S phase in HepG2 cells.

### MCM4, MCM6 and MCM7 maintaining association with chromatin during G2/M phase

**Figure 2:**
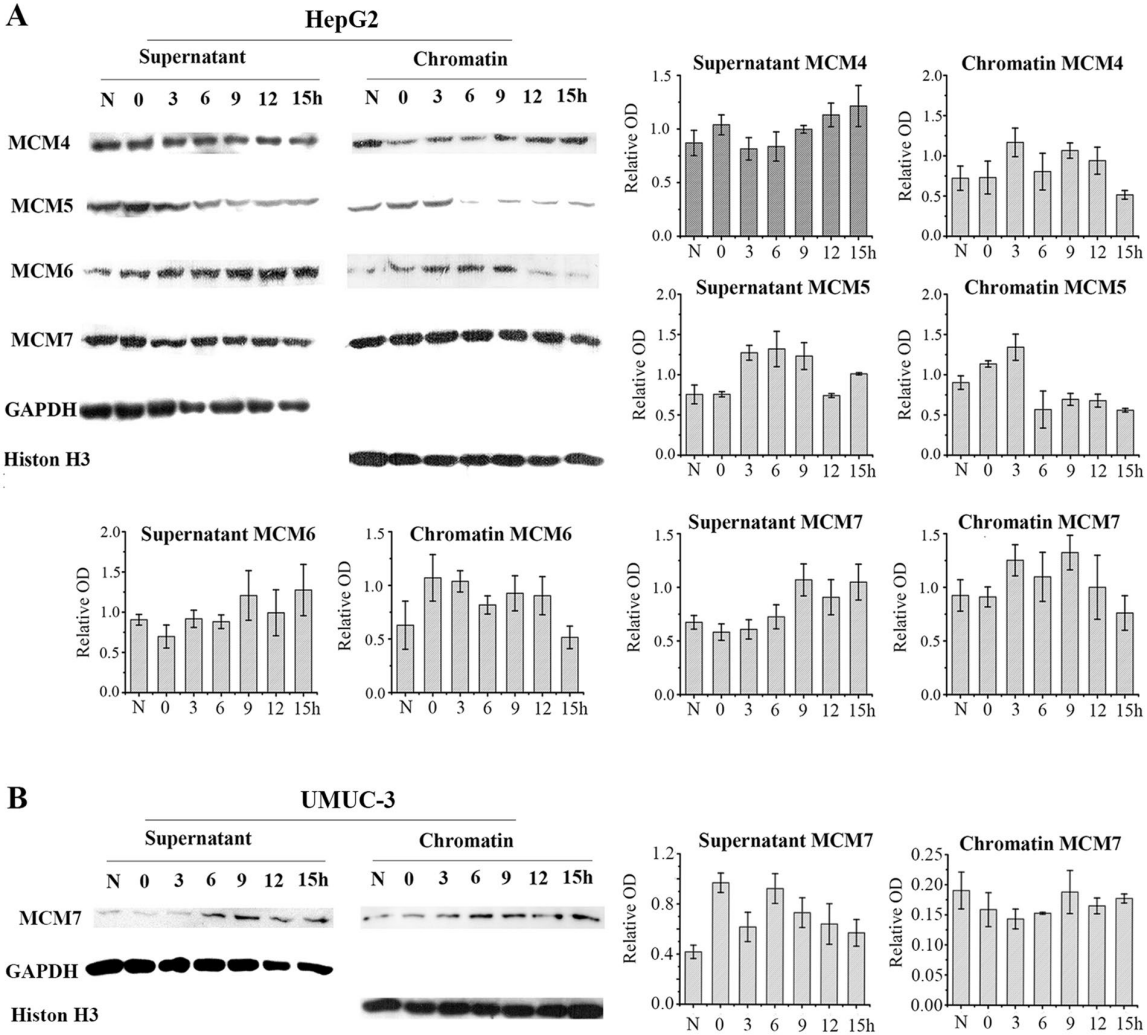
MCM7 keep binding with chromatin in G2/M of HepG2 and UMUC-3 Cells. Cells were treated with double thymine and then incubated in fresh medium for 0 h, 3 h, 6 h, 9 h, 12 h and 15 h. The chromatin binding (chromatin) and non-chromatin binding (supernatant) MCMs in HepG2 cells (**A**) and UMUC-3 cells (**B**) were examined by Chromatin-binding assays. Histone H3 and GAPDH were used as loading control for chromatin-binding proteins and non-chromatin binding proteins respectively. Data are presented as optical density fold difference related to loading control from three independent experiments. N: asynchronized cells.

Chromatin-binding assay were performed to identify distribution of MCMs corresponding to cell cycle progression. In HepG2 cells, chromatin-binding MCM5 were decreased gradually as cells progressed into the G2/M phase. However, large amount of MCM4, MCM6 and MCM7 remained in chromatin in G2/M cells. MCM7 differed from the other MCMs in that it maintained binding to chromatin through the cell cycle (Fig. 2A). In UMUC-3 cells, MCM7 were also observed to be bound to chromatin in G2/M phase (Fig. 2B).

**Figure 3:**
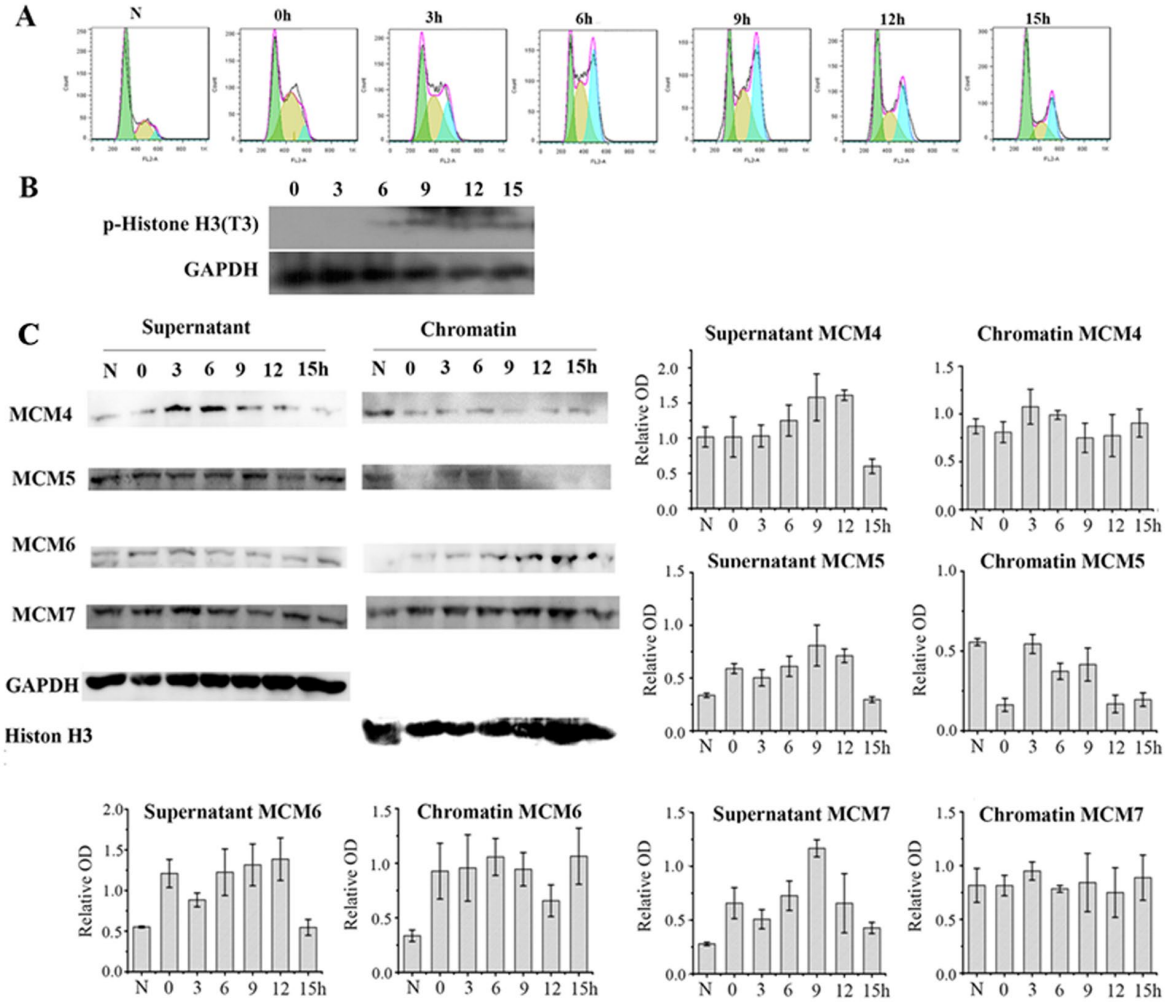
MCM7 constantly bind chromatin throughout cell cycle in SPC-A1 Cells. SPC-A1 Cells were treated with double thymine and then incubated in fresh medium for 0 h, 3 h, 6 h, 9 h, 12 h and 15 h. (**A**) SPC-A1 cell cycle was analyzed by FACS. (**B**) P-Histone H3 was detected by western-blot. (**C**) Chromatin binding and non-chromatin binding MCM4, MCM5, MCM6 and MCM7 were analyzed by chromatin binding assay. Histone H3 and GAPDH were used as loading control for chromatin-binding proteins and non-chromatin binding proteins respectively. Data are presented as optical density fold difference related to loading control from three independent experiments.

Subsequently, we performed the chromatin-binding assay in human lung carcinoma SPC-A1 cells. SPC-A1 cells were synchronized by double thymine block in G1 (G1: 45.23% ± 1.20%). Cells entered G2/M phase after incubation in fresh medium at 6 h (G2/M: 44.51%). Most cells exit M phase after incubation in fresh medium at 9 h (Fig. 3A). The presence of p-Histone was consistent with FACS results (Fig. 3B). Like HepG2 cells, SPA-A1 cells also exhibited sustained MCM7 binding to chromatin through the cell cycle. The chromatin-binding abilities of other MCMs in SPC-A1 cells were similar to that of HepG2 cells, except for MCM5 which was present on chromatin in G2/M SPC-A1 cells (Fig. 3C).

### MCM7 protein present in high level in mitotic cells

**Figure 4:**
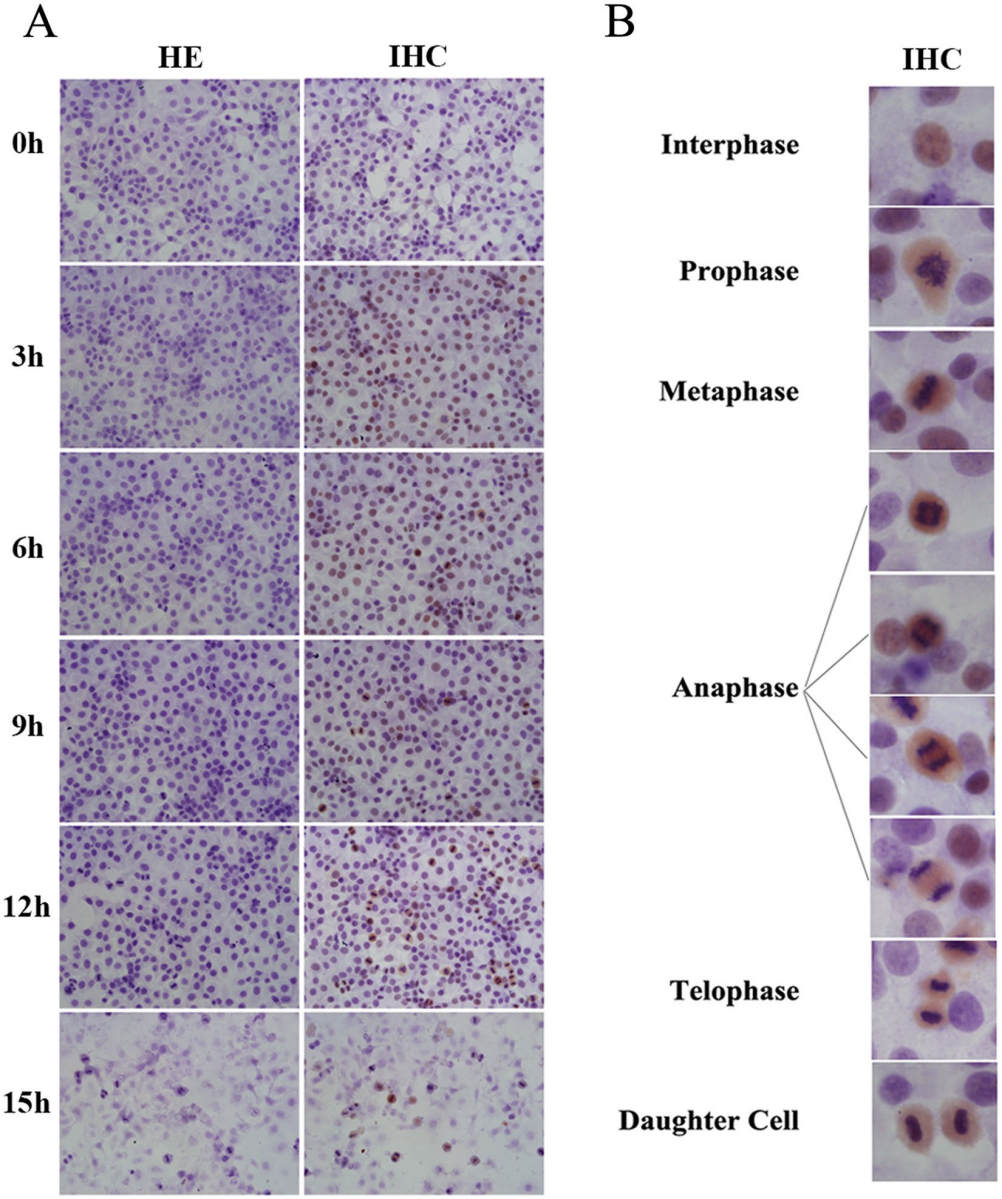
MCM7 proteins highly presents in mitosis phase in HepG2 Cells. (**A**) HepG2 Cells were synchronized by double thymine and then releasing for 0 h, 3 h, 6 h, 9 h, 12 h and 15 h. Western blot analysis of MCM7 (left). Data are expressed as optical density (OD) fold difference related to GAPDH from 3 duplicate experiments (right). N: asynchronized cells. (**B**) and (**C**) Immunohistochemistry analysis of MCM7 in HepG2 Cells. HE: H.E staining, IHC: immunohistochemistry.

To observe MCM7 expression directly in the cells, MCM7 was detected via immunohistochemistry (ICH). MCM7 has high levels of expression throughout the whole M phase, including prophase, metaphase, anaphase and telophase (Fig. 4B).

### MCM7 depletion promotes mitotic exit

**Figure 5:**
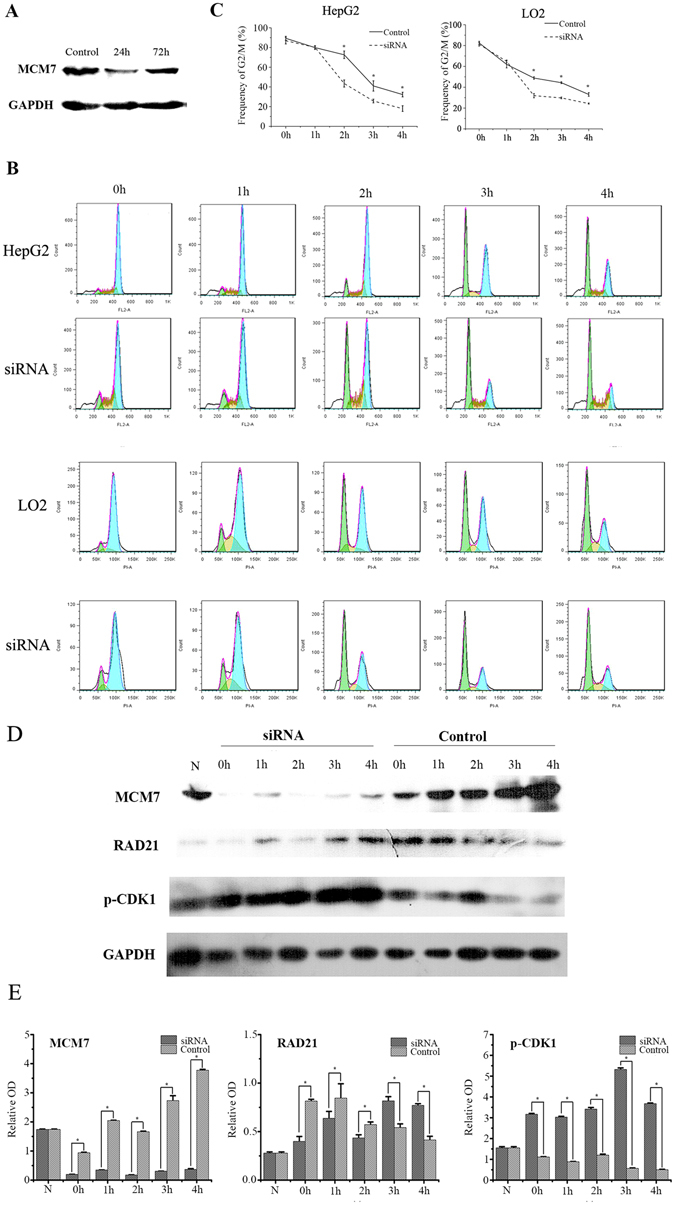
MCM7 depletion promotes mitotic exit. (**A**) HepG2 cells were transfected with MCM7 siRNA or negative control siRNA (Control) for 24 h or 72 h. MCM7 protein level was analyzed by Western blot. GAPDH was used as the loading control. (**B**) HepG2 cells and LO2 cells were transfected with MCM7 siRNA (siRNA) or negative control siRNA (Control) for 12 h, then 0.2 μg/ml nocodazole was added to cell culture for further 12 h and then incubated in fresh medium for 0 h, 1 h, 2 h, 3 h and 4 h. Cell cycle was analyzed by FACS and data are shown as mean ± SD of three independent experiments (**C**), *P < 0.05. (**D**) MCM7, p-CDK1 and RAD21 were detected by western-blot. GAPDH was used as the loading control. Data are expressed as optical density (OD) fold difference related to GAPDH from 3 duplicate experiments (**E**), *P < 0.05.

Based on the latter results, it is confirmed that MCM7 maintains presence and association with chromatin during G2/M phase. To examine its function in the G2/M phase, MCM7 was depleted by RNAi in nocodazole-blocked G2/M cells. After being treated with 0.2 µg/ml nocodazole for 24 h, most HepG2 were blocked in the G2/M phase (G2/M: 89.33% ± 2.01%). After transferring into fresh medium from 0 h to 4 h, cell gradually exited M phase. The RNAi transfection successfully reduced MCM7 expression (Fig. 5A). In the MCM7 siRNA cells, the speed of mitotic exit was quicker than control group. Most cells exited mitosis 2 h after transferring (Fig. 5B,C). To further confirm this phenomenon in no-cancer cells, normal hepatocytes LO2 cells were used to explore MCM7 depletion effect on mitotic progression. The same phenomenon was observed in LO2 cells (Fig. 5B,C).

During M phase, CDK1 inactivation is the key event for activating separase for cleaving Cohesin/RAD21, allowing sister chromatids to segregate. Therefore, we monitored changes in RAD21 and p-Cdk1 levels (p-Cdk1 is a conserved tyrosine phosphorylation (Tyr15 in humans), leading to inhibition of Cdk1 activity). In the control group, after nocodazole release, the p-CDK1 levels were higher in the earlier time points (0, 1, and 2 h) and the RAD21 was gradually cleaved during mitosis exiting. Interestingly, the MCM7 levels were gradually increased. In MCM7 siRNA group, the p-CDK1 levels were kept much higher levels than in the control group indicating that Cdk1 activity was inhibited. Correspondingly, the RAD21 levels were decreased significantly (Fig. 5D,E). These results demonstrated that MCM7 depletion led to inactivation of CDK1 and cohesin/RAD21 degradation.

### MCM7 depletion results in aberrant mitosis

**Figure 6:**
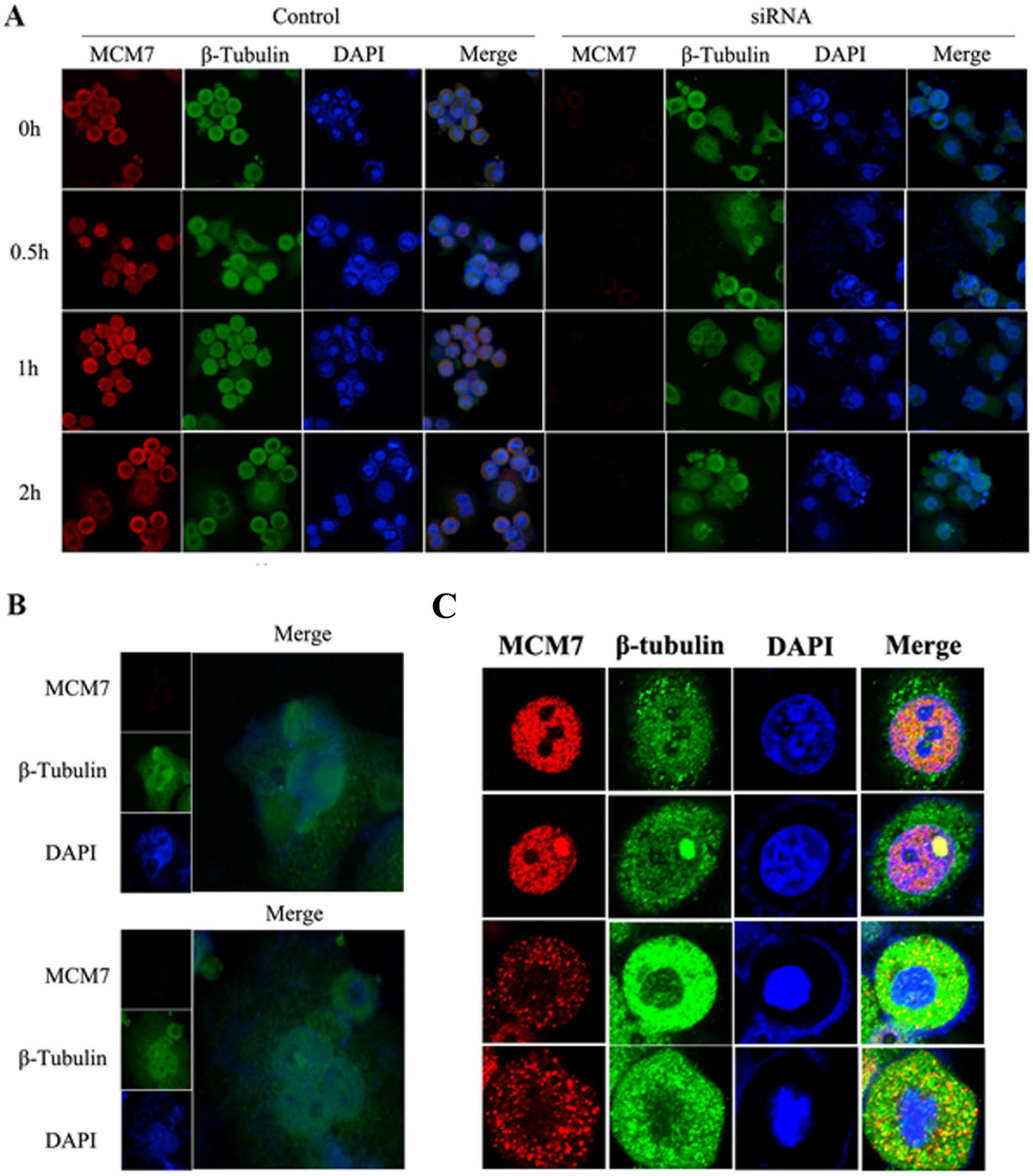
MCM7 depletion contributes to aberrant mitosis. (**A**,**B**) HepG2 cells were transfected with MCM7 siRNA or negative control siRNA. After being transfected for 12 h, cells treated with 0.2 μg/ml nocodazole for further 12 h and then incubated in fresh medium for 0 h, 0.5 h, 1 h and 2 h. Immunofluorescence staining was performed to detect MCM7 (red) and β-Tubulin (green) in the cells, and nucleuses were visualized by DAPI staining (blue). (**C**) HepG2 cells were treated with or without 0.2 μg/ml nocodazole for 12 h to block cell in M phase, above immunofluorescence staining was performed and observed under confocal microscopy.

To further characterize MCM7 in mitosis, HepG2 cells were synchronized by nocodazole and then incubated in fresh medium for 0, 0.5, 1 and 2 h, immunofluorescence was performed to detect the localization of tubulin and MCM7. In the control, cells were retained in mitosis from 0 to 2 h. Compared to control group, cells exited from mitosis at 2 h after MCM7 depletion (Fig. 6A). Moreover, aberrant mitosis was observed in MCM7 depletion groups (Fig. 6B). In the interphase cells, MCM7 was restricted in nuclear. In mitotic cells, when nuclear envelope breaks down, MCM7 was distributed through cell and co-localized with tubulin (Fig. 6C).

## Discussion

In our study, we have found that MCM2-7 mRNA decreased during a cell cycle. More intriguingly, MCM2-7 mRNA was lowest in the G2/M phase. We also found that the reduction was not gradual. MCM2-7 mRNA increased in the S phase and then gradually decreased. This phenomenon was also detected in HeLa cells^23^.

The MCM complex plays an important role in initiating DNA replication in the G1 phase and extending DNA strains in S phase. Therefore, it was assumed that MCMs will disassociate from chromatin in the G2/M phase to avoid DNA reduplication. However, many studies claim that the function of MCMs were not limited to the G1 and S phase. In this research, MCM4, MCM6 and MCM7 were found to be still associated with chromatin in G2/M phase. Previous studies have shown that phosphorylated MCM4 binds to chromatin in G2/M phase in order to prevent MCM complex from regaining association with chromatin to avoid DNA reduplication^24^. Our study is the first to show that MCM6 and MCM7 are still bound to chromatin in the G2/M phase (Figs 2 and 3). The chromatin-bound MCMs’ functions are still unclear. It is possible that MCMs have depolymerized in late G2 phase and there are some MCMs’ subunits still associating with chromatin to regulate M phase events.

To confirm MCM7 expression in a cell cycle, we analyzed protein expression by western-blot and immunohistochemistry (ICH) and demonstrated that MCM7 is always expressed throughout the whole cell cycle. Directly inferring from ICH results, it is surprising that MCM7 have continued expression throughout the entire M phase (Fig. 4). Immunofluorescence results also found that MCM7 maintained expression in mitosis (Fig. 6A).

Next, we determined the function of MCM7 in M phase through the action of MCM7 siRNA. When compared to the negative control, MCM7 depletion accelerated mitotic exit. Moreover, increased CDK1 inhibitory phosphorylation and Cohesin/RAD21 cleavage were observed in MCM7 siRNA group. It is well known that spindle assembly checkpoint (SAC) is the key to control cytokinesis. When spindle assemble completely, SAC will be inactive and ultimately leads to CDK1 inactivity. CDK1 can inhibit separase before anaphase. Separase triggers anaphase by cleaving its substrate cohesin, which is the protein that bind sister chromatids. The inactivation of Cdk1 will activate separase and then trigger cohesin degradation, and ultimately initiates the final separation of sister chromatids^25^. In this research, we demonstrate that MCM7 depletion will lead to CDK1 inactivation and accelerate mitotic exit (Fig. 5). In addition, MCM7 is co-localized with tubulin and MCM7 depletion lead to aberrant mitosis (Fig. 6). Thus, it is possible that MCM7 participates in SAC. MCM7 may help to assemble the spindle and prevent mitosis premature exit. However, the mechanisms underlying MCM7 functions on mitosis events still need further explorations.

Based on this study, we conclude that MCM7 roles are more than just assembling pre-RC complex for launcing DNA replication. MCM7 maintains expression and association with chromatin in M phase to prevent premature exit from mitosis.

## Materials and Methods

### Cell lines

Hepatocellular carcinoma HepG2, human lung carcinoma SPC-A1 and normal hepatocytes LO2 cells were routinely maintained in DMEM medium (Gibco BRL, Grand Island, NY, USA). Human bladder cancer UMUC-3 was routinely maintained in MEM medium (Gibco BRL, Grand Island, NY, USA). The medium contains 10% fetal bovine serum, penicillin (100 U/ml) and streptomycin (100 μg/ml). Cells were cultivated at 37 °C in a balanced air humidified incubator with an atmosphere of 5% CO_2_.

### Cell cycle synchronization

Cells were synchronized at G1/S border by double thymidine block^26^. Briefly, cells were treated with 2 mM thymidine (purchased from Sigma-Aldrich) for 19 h, incubated in fresh medium containing 10% FBS for 9 h and then synchronized for an additional 18 h with 2 mM thymidine. After washing three times with PBS, the blocked cells were incubated in fresh medium containing 10% FBS. Cells were harvested at 0, 3, 6, 9, 12 and 21 h time points. The cell-cycle progression was detected by flow cytometric analysis. For mitosis block, cells were treated with 0.2 μg/ml nocodazole (purchased from Sigma-Aldrich) for 12 h. After washing three times with PBS, the blocked cells were incubated in fresh medium containing 10% FBS. Cells were harvested at 0, 1, 2, 3 and 4 h. The cell-cycle progression was detected by flow cytometric analysis.

### Western blot

Total proteins from cells were extracted using ice-cold lysis buffer (50 mM Tris-HCL pH 7.5, 150 mM NaCl, 1% NP40, 1 mM PMSF, and 10 units/ml aprotinin) for 5–10 min, then centrifuged at 12000 rpm form 10 min at 4 °C to obtain the whole cell lysate. Proteins (about 20 μg) were separated by 12% SDS-PAGE and transferred onto polyvinylidene fluoride membranes and incubated overnight at 4 °C with antibody against MCM4, MCM5, MCM6, and MCM7 (Santa Cruz), p-histone H3 (Thr3) (Cell Signaling technology), histone H3 (Cell Signaling technology), p-Cdk1 (Abcam) or GAPDH (Bioworld technology). After washing with Tris-buffered saline with 0.1% Tween 20, the membranes were incubated with HRP-conjugated IgG at room temperature for 1 h. Signal detection was carried out with an ECL system (Millipore, Billerica, MA, USA). WB band were quantified with gel-pro software and expressed as optical density fold difference related to GAPDH (relative OD).

### Chromatin binding assay

Cells were harvested and resuspended in tubes with EB buffer (100 mM KCl, 50 mM HEPES-KOH pH 7.5, 2.5 mM MgCl_2_, 50 mM Na_4_P_2_O_7_, 0.1 mM NaVO_3_, 0.5% triton X-100) containing protease inhibitors, then set on ice for 5–10 min for incubation. The tubes were flicked to mix the solution every 2–3 min during incubation. Subsequently, 30% ice-cold sucrose containing protease inhibitors was added to the bottom of the tubes. The tubes were then spinned at 12–15 rcf, 10 min, 4 °C and the supernatants were transferred to new tubes. The pellets were washed with EB buffer and flicked to dislodge the pellets from the wall of the tubes and vibrated briefly for resuspension, followed by spinning in a microfuge, 12–15 rcf, 5 min, 4 °C. Combined the supernatants from the two steps (this is the non-chromosomal fraction). The pellets were resuspended with EB buffer (the pellets are the chromatin binding fraction) and analyzed by Western blot.

### Immunohistochemistry

Cells grown on 6-well plates were fixed for 15 min in 4% (w/v) paraformaldehyde (PFA)/PBS after treatment and then permeabilized for 15 min in 0.25% (v/v) Triton X-100/PBS. After fixation and permeabilization, cells were washed three times in PBS and then blocked with goat serum for 1 h. Cells were incubated with antibodies against MCM7 (Santa Cruz) as required for 4 °C overnight, followed by three times wash with PBS and a 60 min incubation with goat anti-mouse HRP-conjugated secondary antibody. Positive signals were visualized using 3, 3′-diaminobenzidine.

### Immunofluorescence

Cells grown on 6-well plates were fixed for 15 min in 4% (w/v) para-formaldehyde (PFA)/PBS after treatment and then permeabilized for 15 min in 0.25% (v/v) Triton X-100/PBS. After fixation and permeabilization, cells were washed three times in PBS and then blocked with goat serum for 1 h. Cells were incubated with antibodies against MCM7, β-Tubulin (Santa Cruz) as required for 4 °C overnight, followed by three times wash with PBS and a 60 min incubation with goat anti-mouse FITC secondary antibody. Fluorescence images were taken under an Olympus fluorescent microscope.

### Flow cytometry

Cells were fixed in 70% ethanol overnight at 4 °C. Fixed cells were then washed once in ice-cold PBS and stained with propidium iodide (PI) staining solution (50 μg/ml PI, 100 μg/ml RNase, 0.05% Triton X-100 in ddH2O) for 20–30 min. PI-stained cells were then analyzed for their DNA content by using FACS (BD biosciences, San Jose, CA, USA).

### SiRNA knockdown

Three Cdc6-targeting siRNAs (si-MCM7: 5′GAGTTGGTGGACTCAATTT3′) were purchased from Guangzhou Ribobiotech. For the transfection of siRNA, cells (5 × 10^5^) were seeded into 6-well plates and then were transfected with siRNA in diluted Lipofectamine containing Opti-MEM Medium (Invitrogen) according to manufacturer’s protocol. Non-targeting siRNA was used as the negative control.

### Statistical analysis

SPSS version 13.0 for Windows was used for all statistical analyses. Average values were expressed as mean ± standard deviation (S.D.) and statistical significance between different groups was determined by the Student’s t-test. Statistical comparisons were made using one-way ANOVA. *P* values < 0.05 were considered to be significant.
